# Supplementary material for: Cloning and Functional Analysis of CsROP5 and CsROP10 Genes Involved in Cucumber Resistance to Corynespora cassiicola
Source: Biology (Basel). 2024 Apr 28;13(5):308. doi: 10.3390/biology13050308 (PMC11117962; doi:10.3390/biology13050308)
Supplement: Supplementary file 1 [file biology-13-00308-s001.zip › Supplement Figure.pdf]

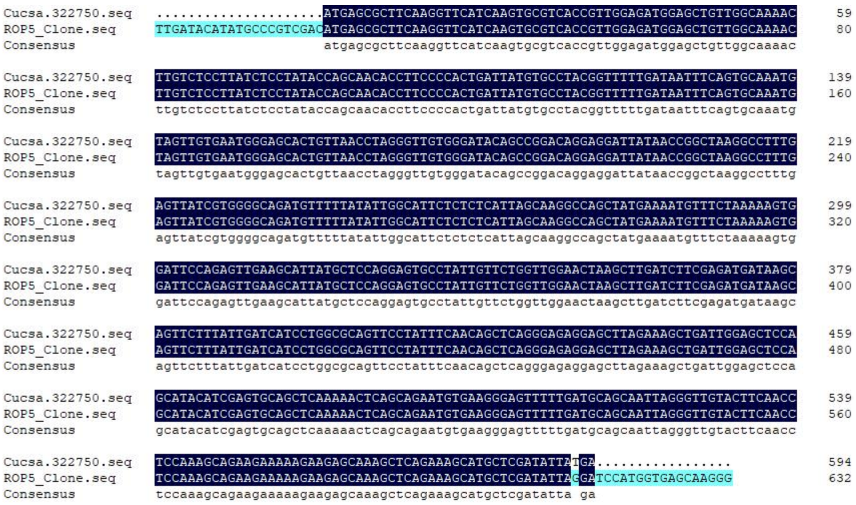

Figure S1 The cloned nucleotide sequence of *CsROP5* cDNA was compared with the known nucleotide sequences in the cucumber genome database.

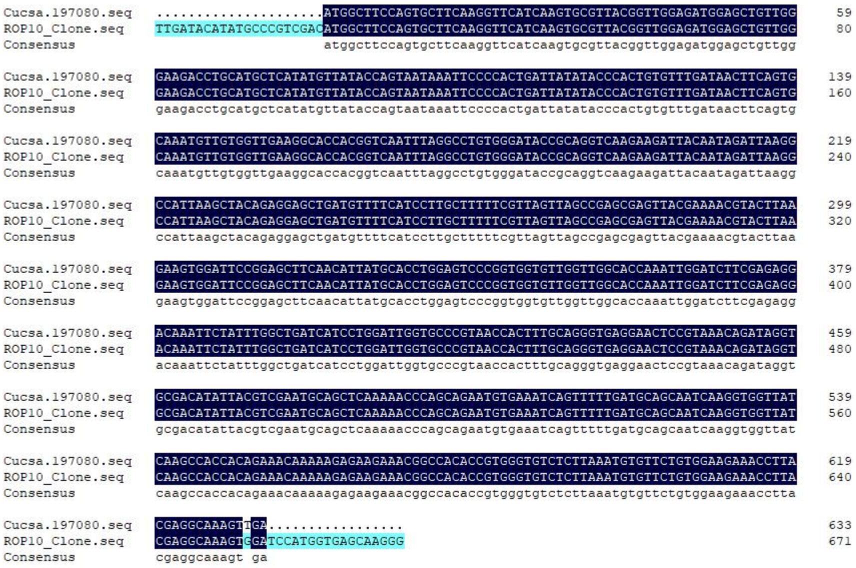

Figure S2 The cloned nucleotide sequence of *CsROP10* cDNA was compared with the known nucleotide sequences in the cucumber genome database.
