# Supplementary material for: Cloning and Functional Analysis of CsROP5 and CsROP10 Genes Involved in Cucumber Resistance to Corynespora cassiicola
Source: Biology (Basel). 2024 Apr 28;13(5):308. doi: 10.3390/biology13050308 (PMC11117962; doi:10.3390/biology13050308)
Supplement: Supplementary file 1 [file biology-13-00308-s001.zip › Supplement Table.pdf]

Table S1 The primers of RT-qRCR used in this study

Analysis	Primer name	Sequence(5′-3′)	Tm/ ℃	Amplicon size/bp
CsPR2 for qRT-RCR in transgenic cucumber	*CsPR2*-F<br>*CsPR2*-R	AAGACAACATCCTCAACTTTGC<br>CTGCTGAAAGAATTCCAGTGTG	58<br>58	192
CsPR3 for qRT-RCR in transgenic cucumber	*CsPR3*-F<br>*CsPR3*-R	CATTTCTCTCATCCTTTGGCAG<br>GATATCGAAATCAACGCCATCC	60<br>60	287
CsPYL2 for qRT-RCR in transgenic cucumber	*CsPYL2*-F<br>*CsPYL2*-R	CAGAAATCATTGATGGACGACC<br>TGAAACATCAGCAAGTGACTTG	60<br>60	143
CsPP2C2 for qRT-RCR in transgenic cucumber	*CsPP2C2*-F<br>*CsPP2C2*-R	TATTCCCGATCCAGAAGTCATG<br>CAAGCATTGAGAGGTAATCTGC	60<br>60	227
CsSnRK2.2 for qRT-RCR in transgenic cucumber	*CsSnRK2.2*-F<br>*CsSnRK2.2*-R	GCAGATCCAGAGACGAGAATAA<br>TCAATACTCTGTGTTGCCTCAT	58<br>58	140
CsABI5 for qRT-RCR in transgenic cucumber	*CsABI5*-F<br>*CsABI5*-R	GAATCGCCATTACAGTCTGAAC<br>AACTCATCCAATGTGAGTGAGT	58<br>58	95
CsRbohD for qRT-RCR in transgenic cucumber	*CsRbohD*-F<br>*CsRbohD*-R	GCGTAACATTACTGGTGATTCC<br>ATTCTTCCATCAGCATCCGTAT	58<br>58	132
CsRbohF for qRT-RCR in transgenic cucumber	*CsRbohF*-F<br>*CsRbohF*-R	ATCGGACGATATTACACAGCTT<br>TTGTACATCAAGCGTCACCT	58<br>58	94
Cucumber *CsActin* gene for and qRT-PCR	Actin-F<br>Actin-R	TCGTGCTGGATTCTGGTG<br>GGCAGTGGTGGTGAACAT	60<br>60	161

Table S2 List of primers used in the study

Analysis	Primer name	Sequence(5′-3′)	Tm	Amplicon size
Sequencing of the cDNA of *CsROP5*	*CsROP5*-F<br>*CsROP5*-R	CATATGCCCGTCGACATGAGCGCTTCAAGGTTC<br>GCTCACCATGGATCCTAATATCGAGCATGCTTTC	60<br>60	591
Sequencing of the cDNA of *CsROP10*	*CsROP10*-F<br>*CsROP10*-R	CATATGCCCGTCGACATGGCTTCCAGTGCTTCA<br>GCTCACCATGGATCCACTTTGCCTCGTAAGGTTTC	60<br>60	630
RT-qPCR for *CsROP5*	*CsROP5*-qF<br>*CsROP5*-qR	TGCTCCAGGAGTGCCTATTGTTC<br>GCTTTCTAAGCTCCTCTCCCTGAG	60<br>60	125
RT-qPCR for *CsROP10*	*CsROP10*-qF<br>*CsROP10*-qR	CGTTAGTTAGCCGAGCGAGTTACG<br>TGTTGAAGCTCCGGAATCCACTTC	60<br>60	61
CsROP5-silencing vector	*CsROP5*-TF<br>*CsROP5*-TR	GTGAGTAAGGTTACCGAATTCGCAGTTCTTTATTGATC<br>GGCCTCGAGACGCGTGAGCTCTAATATCGAGCATGCTTTC	60<br>60	214 bp
CsROP10-silencing vector	*CsROP10*-TF<br>*CsROP10*-TR	GTGAGTAAGGTTACCGAATTCGCAGGGTGAGGAACTC<br>GGCCTCGAGACGCGTGAGCTCACTTTGCCTCGTAAGGTTTC	60<br>60	202
CsROP5-overexpression vector	*CsROP5*-GF<br>*CsROP5*-GR	CATATGCCCGTCGACATGAGCGCTTCAAGGTTC<br>GCTCACCATGGATCCTAATATCGAGCATGCTTTC	60<br>60	591
CsROP10-overexpression vector	*CsROP10*-GF<br>*CsROP10*-GR	CATATGCCCGTCGACATGGCTTCCAGTGCTTCA<br>GCTCACCATGGATCCACTTTGCCTCGTAAGGTTTC	60<br>60	630
Chimeric primer for *CsROP5*-nGFP	*CsROP5-nG*-F<br>*CsROP5-nG*-R	GGGAGTTTTTGATGCAGCAATTAG<br>GGCACGGGCAGCTTGCCGGTGGTG	60<br>60	267
Chimeric primer for *CsROP10*-nGFP	*CsROP10-nG*-F<br>*CsROP10-nG*-R	CCAGCAGAATGTGAAATCAG<br>GGCACGGGCAGCTTGCCGGT	58<br>58	315
